# Microsurgical Debridement for Persistent Ulcers Due to Rare Fungus Infection: Case Report and Literature Review

**DOI:** 10.1055/a-2166-8413

**Published:** 2024-02-07

**Authors:** Yu-Wen Tsui, Chia-Yu Tsai, Hung-Chi Chen

**Affiliations:** 1 Department of Family Medicine, China Medical University Hospital, Taichung, Taiwan; 2 Department of Surgery, China Medical University Hospital, Taichung, Taiwan; 3 Department of Plastic Surgery, China Medical University Hospital, Taichung, Taiwan

**Keywords:** fungal infections, wounds and injuries, *Scopulariopsis brevicaulis*, antifungal agents, microsurgical debridement

## Abstract

A patient suffered from chronic ulcer due to recalcitrant fungal infection for 3.5 years. Five antifungal agents and 40 times of debridement—all failed. Finally, radical microscopic debridement was performed for eradication of fungal conidiospores. Since then, there was no recurrence at 2 years of follow-up. *Scopulariopsis brevicaulis* is one of the rarest pathogens of cutaneous fungal infections, for which multidrug resistance increased the complexity and difficulty of treatment. Radical excision, especially microscopic debridement, was the key for eradication of fungal conidiospores in this case.

## Introduction

Chronic ulcer is a soft tissue damage, which might persist for more than 3 months. When medical treatment of chronic ulcer failed, surgical intervention and even microsurgical debridement, could be considered. Debridement under microscope can radically remove organisms and substantially improve the wound healing. We present an unusual case of a chronic ulcer due to recalcitrant fungal infection. After long-term antifungal medication and debridement for over 40 times, *Scopulariopsis brevicaulis* infection still recurred. Finally, the patient had uneventful recovery after radical microsurgical debridement for fungal conidiospores, with no recurrence at 2 years of follow-up.

## Case

**Fig. 1:**
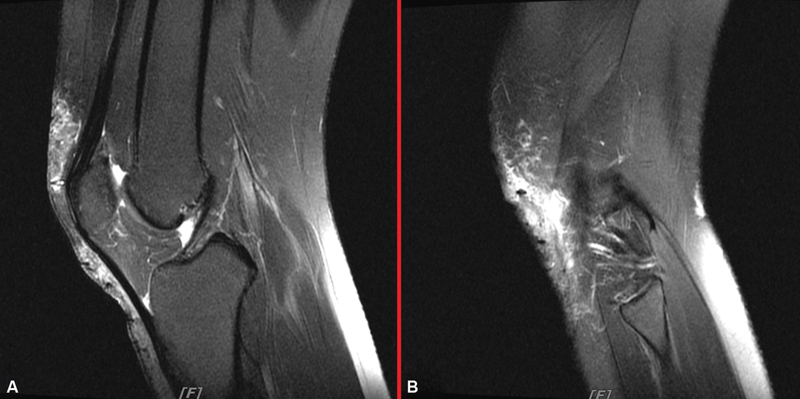
( **A** ) The leg MRI revealed wound infection around right knee with prepatellar bursitis and mild deep infrapatellar bursitis. ( **B** ) Increased fat stranding and heterogeneous enhancement of anterior aspect of right knee, from suprapatellar region to infrapatellar region, compatible with residual cellulitis.

A 30-year-old woman presented with the chief complaint of a laceration wound over the right leg, which resulted from falling into the farm field in a traffic accident. She was sent to a local hospital first, where debridement and wound closure were done. However, deep pus culture of operation sample revealed fungal infection (the species had not been revealed under Gram stain), followed by poor wound healing for months. Recurrent wound infection was noted, as well as pus discharge. So, she underwent debridement and drainage in another local hospital. Nine months after the traffic accident, she was referred to orthopaedic department in our hospital for persistent poor wound healing ( Fig. 1 ). Debridement was done for 11 times, but recurrent fungal infection was still noted. Then, she was referred to infection department. Since repetitive surgeries all failed, as well as antifungal medication with liposomal amphotericin B, flucytosine, and voriconazole, above-knee amputation was even suggested by infection specialist, but the patient refused. Eighteen months after the accident, she was referred to our plastic department.

**Fig. 2:**
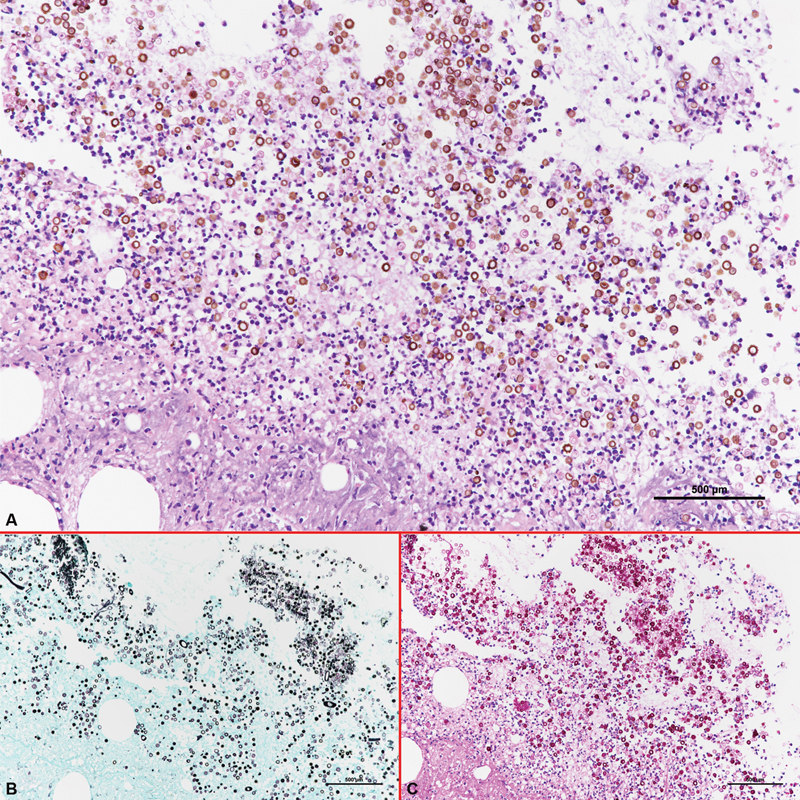
( **A** ) Pathology image of *Scopulariopsis brevicaulis* infection under H&E stain with 20× magnification. The section revealed ulceration with inflamed granulation tissue and abscess formation of subcutaneous tissue. The conidiospores could be seen in a cinnamon-brown cell wall, with long septate hyphae. ( **B** ) Abundant fungal elements are highlighted by GMS stain with 20× magnification. ( **C** ) Abundant fungal elements are highlighted by PAS stain with 20× magnification.

**Fig. 3:**
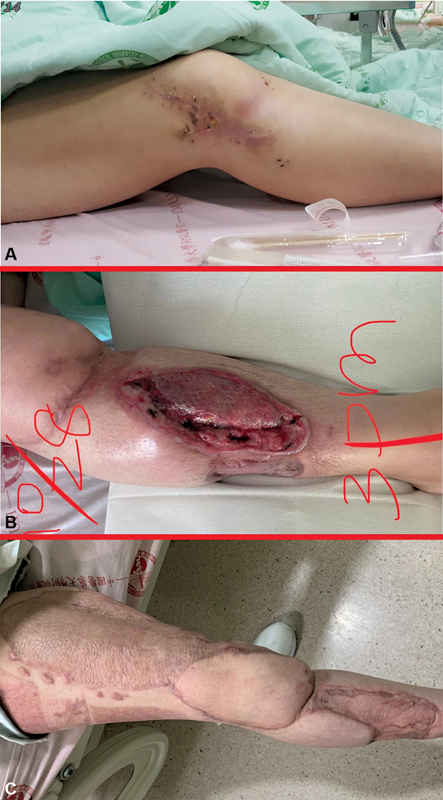
( **A** ) One year after the traffic accident, the right knee chronic ulcer had poor healing with erythematous change and pus formation. ( **B** ) One day after microscopic debridement, fungal elements from the wound were eradicated. ( **C** ) After 3.5 years of debridement, the right leg wound had healed well with no recurrence at 2 years of follow-up.

At our plastic department, she underwent wide excision for four times, followed by fasciocutaneous rotation flap, reverse anterolateral thigh (ALT) flap, and the remaining defect was covered with split-thickness skin graft. Deep pus culture revealed *S* . *brevicaulis* infection. The conidia had a rough wall and a truncated base, mostly in chains. The colonies could be seen with a brown powdery surface and numerous hyaline branched, septate hyphae under a microscope. Then, debridement was done for 19 more times, followed by copious amount of normal saline irrigation with Pulsavac system, accompanied with antifungal medications as micafungin and isavuconazole, etc. However, antifungal medication failed again, and minimum inhibitory concentration results revealed multiple drug resistance ( Table 1 ). Surgical pathology still revealed persistent *S* . *brevicaulis* infection (under Gomori methenamine-silver [GMS] and periodic acid-Schiff [PAS] stain) after 22 months ( Fig. 2 ). Finally, debridement was done under the operating microscope for eradication of fungal elements which appeared brown under magnification and could be easily identified and removed. Since then, excision of adjacent tissues had been done for four times, and deep pus culture no longer revealed fungal infection ( Fig. 3 ). For 3.5 years, the patient suffered from swelling and pain in the chronic ulcers, which exaggerated while walking, and even caused chronic insomnia. She had to quit her job to undergo repetitive operations. Depression was also noted, and she had lost 20 kilograms of body weight altogether. A total of over 40 times of debridement had been done before complete healing. After microsurgical debridement for fungal conidiospores, the patient had uneventful recovery with no recurrence at 2 years of follow-up.

**Table 1:** Minimum inhibitory concentrations (μg/mL) of *Scopulariopsis brevicauli* *s*

ID	*Scopulariopsis brevicaulis* (by Internal Transcribed Spacer [ITS])
Date	April 14, 2021
Method	CLSI-M38A2
Temperature	35°C
Interpretation time	48 hours
Amphotericin B	4
Itraconazole	>16
Voriconazole	16
Posaconazole	>8
Isavuconazole	8
Anidulafungin	0.25
Flucytosine	>8
Terbinafine	>64

## Discussion

According to the data from the World Health Organization, the global prevalence of cutaneous fungal infections is estimated to be approximately 20% of population, and it is rising annually, which is probably due to increasing use of immunosuppressive agents and global travel. 1 Cutaneous and subcutaneous fungal infections may be caused by traumatic injury or through hematogenous seeding (e.g., cryptococcosis, aspergillosis). 2 Among all soft tissue fungal infections, *Scopulariopsis* spp. is one of the rarest fungi, commonly isolated from moist environments. Besides soft tissue infection, fungus sometimes causes keratitis after eye trauma, or pneumonia, brain abscess, endocarditis in immunosuppressed patients. 3

Diagnosis of *Scopulariopsis* is difficult since clinical manifestation is indistinguishable from other fungi like *Aspergillus* . Therefore, histopathological examination can be helpful. Cultures, including blood culture, are usually negative. 4 In an 11-case study, only 7 cases were reported positive in blood culture. Polymerase chain reaction has been recently developed to detect the 28S large-subunit ribosomal RNA gene targeting but has not been widely used for patients. 5 Detection of 1,3-b-D-glucan—a cell wall component of *Scopulariopsis* , can also be useful—is not available in Taiwan. 6 To date, the best treatment remains unknown since *Scopulariopsis* is reported to be resistant to amphotericin B, flucytosine, fluconazole, itraconazole, and miconazole. Moreover, its multidrug resistance might be intrinsic because 84.4% of the cases had no history of antifungal treatment in a 32-case study. 7 Therefore, radical excision of necrotic tissue should be considered while medical treatment is in vain. However, conidia of *Scopulariopsis* are refractory to normal debridement since they are nearly invisible to the naked eye. If debridement has also failed for several times, radical microscopic debridement should be taken into consideration, performed under the microscope for eradication of fungal elements. Microsurgical debridement may require a skilled microsurgeon, advanced equipment, ample operation time, and is done under general anesthesia. In such circumstances, it can be the final solution for the chronic ulcer.
